# Comparison of non-contrast fresh blood imaging and quiescent inflow single-shot imaging at abdominal/pelvic station at 3T

**DOI:** 10.1186/1532-429X-16-S1-P174

**Published:** 2014-01-16

**Authors:** Xiangzhi Zhou, Mitsue Miyazaki

**Affiliations:** 1 Toshiba Medical Research Institute USA, Vernon Hills, Illinois, USA

## Background

Despite increasing application of non-contrast techniques Fresh Blood Imaging (FBI) and Quiescent Inflow Single-Shot (QISS) in the evaluation of peripheral arterial disease (PAD), imaging of abdominal/pelvic station remains a challenge. B0 and B1 inhomogeneity, motion artefacts and poor ECG trigger may diminish the image quality and reduce the diagnostic performance. In this study, we intend to compare the performance of the two non-contrast MRA techniques at the pelvic station at 3T and evaluate their MRA image quality.

## Methods

The study was approved by our institutional review board and informed consent was obtained. Seven healthy volunteers (28-52 years; 1 female) and one patient (65 years; male) were enrolled and scanned by a 3T scanner (Toshiba Vantage) equipped with atlas spine coil and atlas body coil. Followed by the localizer, pelvic station was imaged using FBI and QISS sequences with ECG gating. FBI parameters: 3D coronal single-shot half Fourier FSE, TR = 3RR, TE = 30 ms, 64-80 slices, slice thickness = 3 mm, matrix 256 × 256; FOV 37 cm × 37 cm, parallel imaging factor = 2, flip/flop angle = 90°/140°, TDsys = 0.215RR, TDdias = 0.855RR, Resolution 1.4 mm × 1.4 mm. QISS parameters: single shot bSSFP, TR/TE = 3.4/1.7 ms, 100-120 slices for one station scan (60-80 slices/station if two stations were scanned), slice thickness = 4.0 mm, slice gap = -1.0 mm, matrix: 128 × 256, FOV 32 cm × 39 cm, parallel imaging factor = 3, flip angle = 85°, double fat sat, TD = 100 ms, QI = 220-320 ms, walking sat offset = 5.2 cm, walking sat thickness = 10 cm, one slice/RR, refine in RO and PE direction. Overall image quality and artery quality at 4 segments (abdominal aorta, common iliac artery (CIA), internal iliac artery (IIA), and external iliac artery (EIA)) were blindly scored by 2 experienced clinical scientists (0: low, 4: high). Student t-test was performed to compare the image/segment quality.

## Results

The FBI image quality at pelvic station is significantly higher than the QISS images. In terms of venous signal and background suppression, FBI shows significantly less contamination than QISS. The abdominal aorta, CIA, IIA, EIA segments have significantly better delineation in FBI images than in QISS images. In the patient with stenosis on iliac arteries, FBI MIP images clearly delineate the stenosis level at different stenosis sites.

## Conclusions

Although it is reported QISS may have advantages over FBI in terms of image quality at 1.5T, in this study, we obtained opposite results at pelvic station at 3T. Possible reasons are: QISS is sensitive to susceptibility artefacts; B0 and BI inhomogeneity may have stronger impact on QISS images and led to more residual signal from background tissue and venous blood; TD, QI and walking saturation pulse may not be optimized. Compare to QISS, FBI doesn't require shimming, has higher resolution in the Z direction; is not sensitive to improper ECG trigger. This is a relatively small study; more patient data needs to be collected to further evaluate the performance of FBI and QISS at 3T.

## Funding

NA.

**Figure 1:**
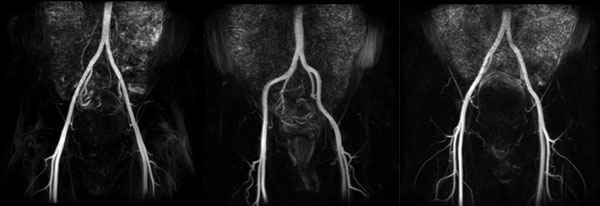
**Iliac MRA images acquired by FBI from three healthy volunteers**. The coronal MIP images shows consistent arterial quality across all volunteers.

**Figure 2:**
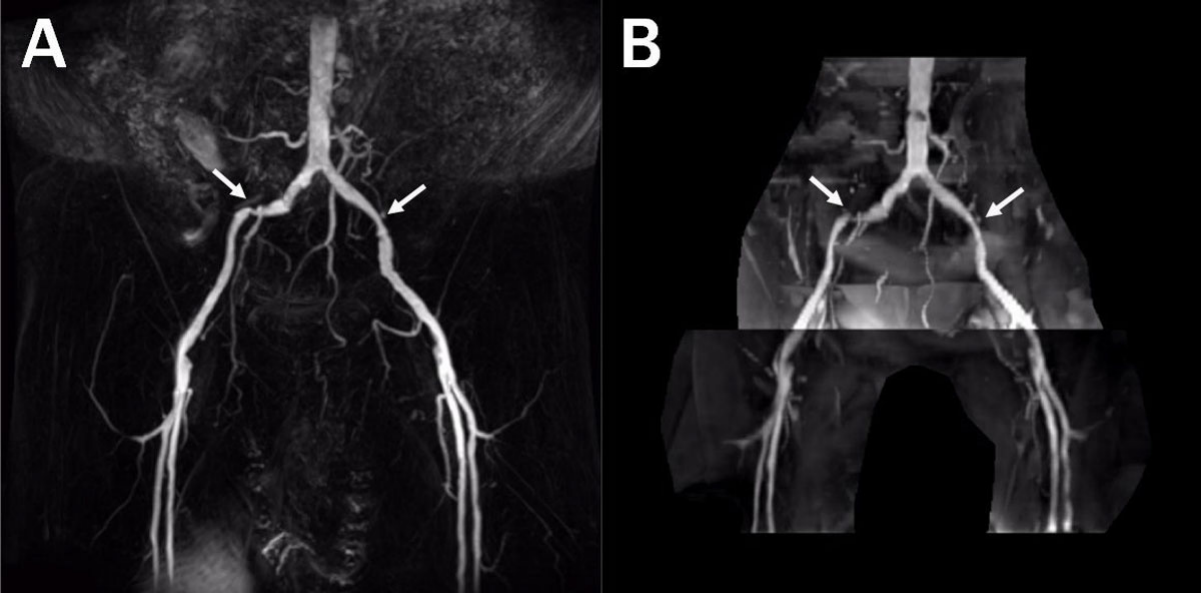
**MIP images at pelvic station acquired by FBI (A) and QISS (B) from one patient with stenosis on both iliac arteries**.

